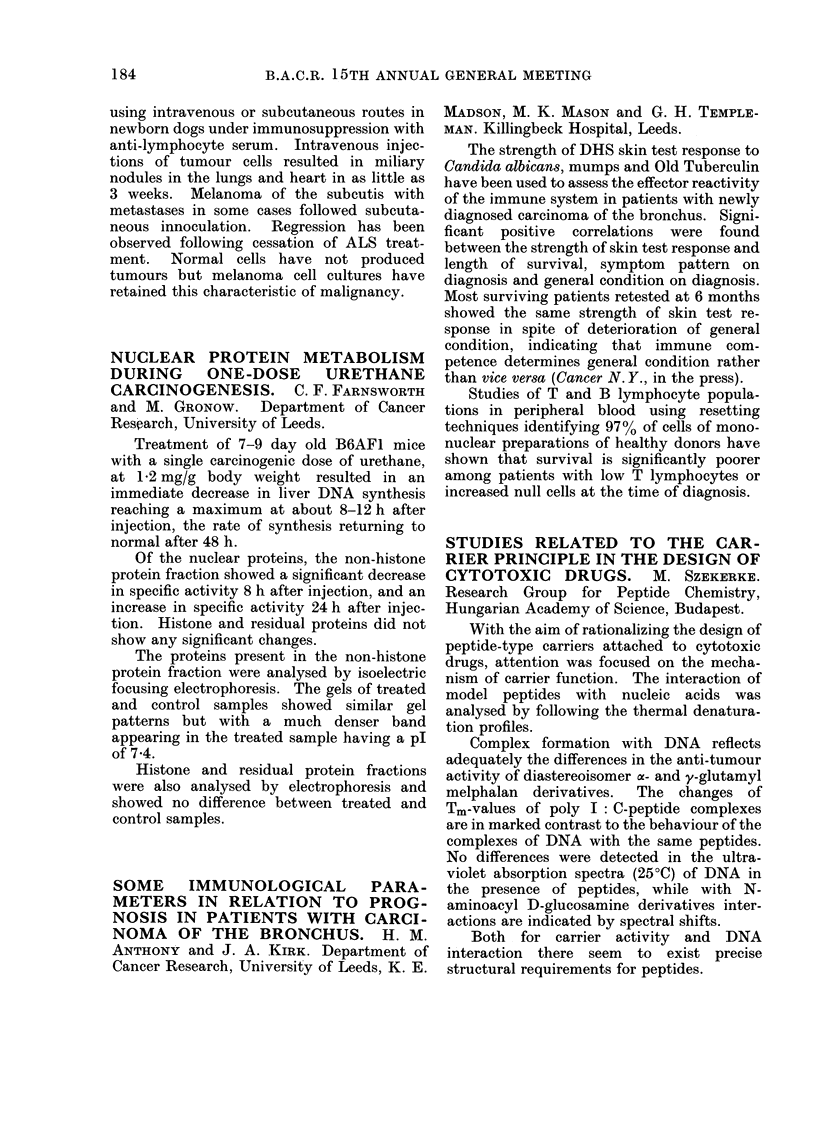# Proceedings: Studies related to the carrier principle in the design of cytotoxic drugs.

**DOI:** 10.1038/bjc.1974.171

**Published:** 1974-08

**Authors:** M. Szekerke


### STUDIES RELATED TO THE CARRIER PRINCIPLE IN THE DESIGN OF CYTOTOXIC DRUGS.

M. Szekerke. Research Group for Peptide Chemistry, Hungarian Academy of Science, Budapest.

With the aim of rationalizing the design of peptide-type carriers attached to cytotoxic drugs, attention was focused on the mechanism of carrier function. The interaction of model peptides with nucleic acids was analysed by following the thermal denaturation profiles.

Complex formation with DNA reflects adequately the differences in the anti-tumour activity of diastereoisomer $\alpha$- and $\gamma$-glutamyl melphalan derivatives. The changes of $T_m$-values of poly I : C-peptide complexes are in marked contrast to the behaviour of the complexes of DNA with the same peptides. No differences were detected in the ultraviolet absorption spectra (25°C) of DNA in the presence of peptides, while with N-aminoacyl D-glucosamine derivatives interactions are indicated by spectral shifts.

Both for carrier activity and DNA interaction there seem to exist precise structural requirements for peptides.